# Enteric Pathogens in Stored Drinking Water and on Caregiver’s Hands in Tanzanian Households with and without Reported Cases of Child Diarrhea

**DOI:** 10.1371/journal.pone.0084939

**Published:** 2014-01-02

**Authors:** Mia Catharine Mattioli, Alexandria B. Boehm, Jennifer Davis, Angela R. Harris, Mwifadhi Mrisho, Amy J. Pickering

**Affiliations:** 1 Environmental and Water Studies, Department of Civil and Environmental Engineering, Stanford University, Stanford, California, United States of America; 2 Woods Institute for the Environment, Stanford University, Stanford, California, United States of America; 3 Ifakara Health Institute, Bagamoyo Research and Training Unit, Bagamoyo, Tanzania; University of Ottawa, Canada

## Abstract

**Background:**

Diarrhea is one of the leading causes of mortality in young children. Diarrheal pathogens are transmitted via the fecal-oral route, and for children the majority of this transmission is thought to occur within the home. However, very few studies have documented enteric pathogens within households of low-income countries.

**Methods and Findings:**

The presence of molecular markers for three enteric viruses (enterovirus, adenovirus, and rotavirus), seven *Escherichia coli* virulence genes (ECVG), and human-specific *Bacteroidales* was assessed in hand rinses and household stored drinking water in Bagamoyo, Tanzania. Using a matched case-control study design, we examined the relationship between contamination of hands and water with these markers and child diarrhea. We found that the presence of ECVG in household stored water was associated with a significant decrease in the odds of a child within the home having diarrhea (OR = 0.51; 95% confidence interval 0.27–0.93). We also evaluated water management and hygiene behaviors. Recent hand contact with water or food was positively associated with detection of enteric pathogen markers on hands, as was relatively lower volumes of water reportedly used for daily hand washing. Enteropathogen markers in stored drinking water were more likely found among households in which the markers were also detected on hands, as well as in households with unimproved water supply and sanitation infrastructure.

**Conclusions:**

The prevalence of enteric pathogen genes and the human-specific *Bacteroidales* fecal marker in stored water and on hands suggests extensive environmental contamination within homes both with and without reported child diarrhea. Better stored water quality among households with diarrhea indicates caregivers with sick children may be more likely to ensure safe drinking water in the home. Interventions to increase the quantity of water available for hand washing, and to improve food hygiene, may reduce exposure to enteric pathogens in the domestic environment.

## Introduction

Over 6.5 million children died in 2012 before reaching their fifth birthday [1]. Globally, almost 10% of these deaths are attributed to diarrhea, and the highest rates of child mortality occur in sub-Saharan Africa [1]. The east African country of Tanzania is a nation that continues to struggle with the burden of childhood diarrhea. In 2010, diarrheal diseases were responsible for almost 9% of all deaths of Tanzanian children under the age of five, just behind malaria (11%) and pneumonia (15%) [2]. Diarrhea-causing pathogens are transmitted via the fecal-oral route and, in low-income countries like Tanzania, it has been suggested that up to 88% of all child diarrhea cases can be attributed to inadequate sanitation, unsafe water, and/or insufficient hygiene [3]. The large diarrheal burden in Tanzania is consistent with the fact that only half of the Tanzanian population has access to improved drinking water sources, and only 10% has access to improved sanitation [4].

Fecal contamination is traditionally monitored using fecal indicator bacteria (FIB) characteristic of animal and human feces (*e.g., Escherichia coli*, enterococci, and fecal coliforms). The concentration of these organisms is regularly used to evaluate health risk associated with different exposure pathways, such as recreational water use and drinking water [5]. For young children, exposure to feces is thought to occur primarily within the home [6], and previous research in low-income countries has documented high levels of FIB in household stored drinking water [7]–[9] and on hands of mothers [10]–[13].

Several studies have investigated the behavioral determinants of FIB levels in stored water and on hands in order to identify the most promising household-level interventions for preventing childhood diarrhea. Safe storage containers and point-of-use (POU) treatments have been found to significantly reduce FIB contamination of household stored drinking water [14], [15]. Other studies investigating hand contamination found that increasing the frequency of hand washing with soap reduces FIB contamination on hands, whereas some household chores increase hand contamination [16], [17].

Several studies have found associations between FIB on hands and/or in stored drinking water and diarrheal illness in children [18]–[20]. For example, one study in the Philippines found that children drinking water with high levels of *E. coli* had significantly higher rates of diarrhea than those drinking less contaminated water [19], and a recent study in Tanzania found FIB contamination on hands to be significantly associated with diarrheal illness among household members [17]. However, other research suggests that FIB are inadequate indicators of the health risks associated with fecal contamination in the home [21]–[23]. For example, in Ecuador Levy *et al.* found childhood diarrhea to be associated with levels of *E. coli* in drinking water, but not with the concentration of enterococci or somatic coliphage [24]. Similarly, a meta-analysis by Gundry *et al.* was unable to determine any clear relationship between FIB levels in household drinking water and incidence of child diarrhea [22].

One possible reason for the equivocal findings regarding the relationship between FIB and child diarrhea is that FIB are present in both human and non-human animal feces [5]. In addition, FIB have historically been used to assess water quality in countries with temperate climates. They have subsequently been found to occur naturally and even proliferate in water [25], [26], soil [27]–[29], and sands [30], particularly in tropical environments [31], [32]. As such, FIB in water and on hands are often not strongly associated with the presence of human enteric pathogens [33]–[36], and there is very limited research on the association between indicators and pathogens in South America, Asia, and Africa [37]. In particular, viruses such as human enterovirus, adenoviruses, and rotavirus show limited association with FIB [33], [37]–[43]. Given the low infectious dose of these viruses, as well as their significant contribution to child diarrhea globally, the limited utility of FIB to indicate viral presence in water and on hands is of particular concern [44], [45].

For these reasons, in low-income countries with widespread fecal contamination, FIB are likely not adequate indicators for understanding the relationship between water management and hygiene behaviors and diarrheal illness among young children. Molecular fecal markers such as human-specific *Bacteroidales* that are unique to human fecal sources [46], as well as molecular markers of enteric pathogens, may be better indicators of contamination. In turn, such indicators may be more useful for identifying behavioral interventions that can prevent childhood diarrhea. However, few studies have reported the prevalence of enteric pathogens or human-specific fecal contamination in the household environment of low-income countries [16], [33], [47].

In a recent study conducted in Bagamoyo, Tanzania, Mattioli *et al*. analyzed source water, stored drinking water, and hand rinse samples from 93 households for molecular markers of enteric viruses and *E. coli* virulence genes [33]. The authors found a significant association between viral markers on hands and in stored drinking water, as well as between *E. coli* virulence genes in stored and source waters. FIB were found not to be good predictors of viral markers in water or on hands, although turbidity was associated with viral markers on hands. However, Mattioli *et al*. did not examine the association between microbial contamination and either child health or water management and hygiene behaviors among sampled households.

The present study builds on Mattioli *et al*. [33] by using data collected from 223 households in Bagamoyo, Tanzania to investigate whether the presence of enteropathogen and human-specific *Bacteroidales* genes in water and on hands is associated with reported cases of child diarrhea. Of the 223 households, 213 households are distinct from those described previously [33]. In addition, we combine the microbial data collected uniquely for this study with data from the 93 households described in Mattioli *et al*. [33] in order to evaluate the association of water management and hygiene behaviors with enteropathogen and human-specific *Bacteroidales* genes in stored drinking water and hand rinse samples.

## Methods

### Setting

The study was conducted in Bagamoyo, Tanzania (06°28′S, 38°55′E), approximately 70 km north of Dar es Salaam. Households–defined as groups of people that sleep and eat together in a dwelling on a regular basis–that included at least one child under five years of age were enrolled in the study. The data used in the present study were collected from a subset of 1219 households surveyed during the baseline phase of a household water and hygiene behavioral intervention trial from March-May 2010 [33].

### Data Collection

In each participating household, a hand rinse sample was taken from the respondent (adult female caregiver). Hand rinse sampling involves the participant placing her hands, one at a time, into a sterile sample bag containing 350 ml of sterile distilled water, a sampling method used successfully in a number of previous studies [13], [16], [17], [33]. A sample of stored water that was intended for drinking and cooking was also collected from each household. Enumerators documented whether or not the water storage container was covered, then asked each respondent to extract water in the manner she usually would, pouring it into a 1.63 liter sterile sample bag (VWR, Radnor, PA). Enumerators noted whether the respondent’s hand touched the water during extraction, as well as the extraction method used (*e.g.,* decanting, filling a cup or bowl). Enumerators inquired whether and how the water had been treated, and for how long the water had been stored prior to sampling. Finally, respondents were asked to identify the water source from which the stored water had been collected. Water samples were tested for chlorine using a dip chlorine strip (Hach Co., Loveland, CO); because chlorine was never detected, there was no need to add sodium thiosulfate to neutralize residual.

Along with water and hand rinse samples, enumerators conducted interviews with the female caretaker of the youngest child in the household. Information was collected regarding household water management and hygiene behaviors, water supply and sanitation services, household socioeconomic and demographic characteristics, and illness status of household members. Tanzanian enumerators participated in a 4-week training that included instruction on survey content and administration, electronic data collection, and sterile sampling technique. The survey instrument underwent multiple iterations and pre-tests.

### Ethics Statement

Participants were informed in the local language (Kiswahili) of all study procedures and the time required for participation. Written informed consent was obtained from the mother or primary female adult caretaker of the under-five children in the household. When younger caregivers were interviewed (15–17), an adult household member was present. The Tanzanian Commission for Science and Technology, the Tanzanian National Institute for Medical Research (NIMRI) Ethics Sub-Committee, the Ifakara Health Institute Institutional Review Board (IRB), and Stanford University’s IRB (IRB Protocol #17971) approved the consent procedures and study protocol.

### Laboratory Analysis

All water and hand rinse samples were stored in a cooler on ice and transported to a local laboratory for microbial analysis by membrane filtration within six hours of collection. The turbidity of the water and hand rinse samples was measured using a LaMotte 2020e Turbidity Meter (LaMotte Company, Chestertown, MD). The fecal indicator bacteria, *E. coli* and enterococci, were enumerated following USEPA Methods 1604 and 1600, respectively [48], [49]. MgCl_2_ was added to the water and hand rinse filters to facilitate capture of viral particles [50], and the samples were subsequently passed through a 0.45 µm-pore size membrane filter as outlined in Mattioli *et al*. [33]. The filters were then treated with RNAlater (Qiagen, Germantown, MD) to stabilize RNA/DNA [51] and stored at −80°C. *E. coli* membrane filters with *E. coli* biomass (from EPA method 1604) were removed from agar after counting and were treated with RNAlater and stored at −80°C. Filters were stored for up to 5 months at −80°C until being transported back to Stanford University (Stanford, CA, USA) for molecular processing. Details on field and lab blanks, turbidity measurements, filtration volumes, culture assay detection limits, and sample transport were previously described by Mattioli *et al*. [33].

The presence or absence of the nucleic acids from three enteric viruses (enterovirus, adenovirus, and rotavirus), the human-specific *Bacteroidales* marker, as well as seven *E. coli* virulence genes was measured in all samples. These pathogens were chosen for analysis because rotavirus, pathogenic *E. coli*, and *Shigella* spp. are believed to be major viral and bacterial etiologies of childhood diarrhea [52], [53]; in addition, enteroviruses and adenoviruses are recognized as important etiological agents of gastroenteritis for children in the developing world [45], [54].

Presence of *E. coli* virulence genes in preserved *E. coli* biomass was determined using multiplex polymerase chain reactions (PCR) [33]. This method determines the presence of diarrheagenic *E. coli* virulence genes that are commonly found in *Shigella* spp., as well as five different pathotypes of *E. coli* including enteroinvasive *E. coli* (EIEC), enteropathogenic *E. coli* (EPEC), enteroaggregative *E. coli* (EAEC), enterotoxigenic *E. coli* (ETEC), and enterohemorragic *E. coli* (EHEC) [47]. These virulence genes include *stx1* and *stx2* (present in EHEC), *eaeA* (present in EHEC and EPEC), *STIb* and *LTI* (present in ETEC), *ipaH* (present in EIEC and *Shigella* spp.), and *aggR* (present in EAEC) [55].

The three enteric viruses (enterovirus [56], rotavirus [57], and adenovirus [58]) and the human-specific *Bacteroidales* fecal marker (BacHum) [59] were detected using end-point PCR (BacHum) or reverse transcriptase-PCR (viruses) with a hydrolysis probe. Details on nucleic acid extraction, molecular detection assays, assay detection limits, and inhibition analyses can be found in Mattioli *et al*. [33].

### Statistical Analysis

Data were analyzed using SAS Enterprise Guide version 4.3 (SAS Institute Inc., Cary, NC). The term ‘enteric virus’ is defined as the presence or absence of at least one of the three enteric viruses measured. The acronym ECVG is defined as the presence or absence of at least one of the seven *E. coli* virulence genes measured, and the term BacHum is defined as the presence or absence of the human-specific *Bacteroidales* fecal marker. Results are considered statistically significant at a level of p≤0.05.

A matched case-control study design was used to evaluate the relationship between cases of diarrhea in children under five and the presence of molecular markers of enteric pathogens on hands of mothers and in household stored water. Out of the 1219 households surveyed in the larger household water and hygiene behavioral intervention trial, the total number of children classified as sick with GI illness was 113 (among 112 unique households). These children served as our ‘cases’ in the case-control study. A case of gastrointestinal illness (GI) was defined as a child having three or more loose/watery stools per 24 hour period, blood in the stool, and/or vomiting using a two-day recall period. Reported GI illness was thus nearly contemporaneous with the sampling and interview. Healthy children (no reported symptoms) in the same household as a GI case were excluded from being controls. Children presenting with non-GI symptoms (*e.g*., coughing, congestion) and their siblings were also excluded from being controls.

Cases were matched to controls post sample collection by one-to-one propensity score matching (PSM) with no replacement using STATA (version 11; Stata Corporation, College Station, TX) [60]. PSM was employed to reduce the chance of bias resulting from systematic differences between cases and controls and to improve effect estimation efficiency [61]. The following variables were included in the model to generate the propensity scores for matching: child age; the number of families in the housing unit; the number of times mother reported washing hands with soap the day prior to interview; whether the household was located in an urban or rural community; whether the mother reported that the youngest child uses a latrine regularly; whether the household has an on-plot water source; if the child’s palms were observed to have visible dirt; whether the mother works outside the home; the number of liters of water collected *per capita* per day; and whether the household’s main drinking water source type was a borewell or tap. An equal number of children (among 111 unique households) were selected by PSM as matched controls.

Conditional logistic regression was used to calculate matched odds ratios (OR) representing the association between childhood diarrhea and the presence of contamination on the primary caregiver’s hands and in the household’s stored drinking water (case-control analysis). Exact p-values and confidence intervals (CI) were calculated. As a robustness check and to control for potential confounders in the case-control analysis, bivariate analyses of household demographics and water, sanitation, and hygiene behaviors/characteristics between case and control households were performed. The bivariate analyses showed which factors were independently associated with child case status. The student’s T-test was used to compare mean values between case and control households. Pooled variances were used unless the Equality of Variances Folded F-Statistic was significant (*p*<0.05), in which case a Satterthwaite Test of unequal variance was used. The chi-square test was used to test for differences in proportions of binary variables between case and control households; when N <5 a Fisher's exact test was used. An ANOVA test was used to evaluate differences in proportions between case and control households for categorical variables, and the Wilcoxon-Mann-Whitney Test was used for non-parametric comparisons for non-normally distributed continuous variables. Variables with a p-value ≤0.20 in the bivariate analysis were included in a multivariate logistic regression model of diarrhea. Model reduction was performed by backwards selection until all remaining variables were found to be significant (*p*≤0.05). Adjusted odds ratios (AOR) and 95% confidence intervals were calculated using conditional logistic regression, adjusting for those variables independently associated with diarrhea [62]. The possibility of overmatching with PSM was also assessed and is described in the Supporting Information (SI).

Multivariate logistic regression was used to model the presence of contamination (separate models were estimated for ECVG, enteric virus, and BacHum) on hands and in household stored water as a function of hand hygiene and water management behaviors, respectively. Each separate model included data from 306 unique households. Two hundred twenty-three of these households were part of the case-control analysis presented above. The additional 83 households were drawn from the dataset published by Mattioli *et al*. [33].

The hand hygiene behaviors examined in the model of hand contamination include reported liters *per capita* per day used for hand washing; whether the respondent reported washing her hands with soap within one hour prior to having her hand rinse taken; and the respondent’s reported activity immediately prior to having the hand rinse sample taken (*i.e.,* washing clothes, dishes, or child; hand washing; food preparation/eating/serving; or other activity such as gardening/farming and sweeping; *versus* sitting).

The water management behaviors examined in the model of stored drinking water contamination include whether the respondent reported actively treating her stored water (*i.e.,* boiling, chlorinating, filtering, or solar water disinfection); the reported length of time the sampled water had been stored in the home; whether the observed extraction method of stored water for sampling by the respondent was “risky” (*i.e.,* dipping a short-handled cup, mug, or bowl); whether the stored drinking water was reportedly collected from an improved source; the average reported amount of time that household members spend fetching water per day; and whether the bacterial or viral gene(s) being modeled was present in the respondent’s hand rinse sample. The models also include control variables for the presence of an infant in the household, household monthly expenditure *per capita*, and whether the household uses a facility with improved sanitation infrastructure. Other control variables considered but that did not contribute significantly to any of the models include whether the household used a private or shared latrine; the number of children under five in the household; whether the primary caregiver worked outside of the home, and whether the primary caregiver was able to read and write.

For the purposes of this manuscript, improved sanitation infrastructure is defined as a toilet or latrine with a cement slab, septic tank, or flush tank into a piped sewer system or pit latrine. This definition differs from the WHO/UNICEF Joint Monitoring program definition [63] in which only privately owned sanitation facilities are considered improved. This definition allowed us to examine for the effects of private versus shared sanitation and improved versus unimproved sanitation infrastructure separately in our models.

## Results

### Matched Case-control Analysis

Among the 113 children classified as sick with GI illness within the 48 hours prior to interview, 80 children were reported to have 3 or more loose/watery stools per 24 hour period, 6 were reported to have blood in the stool, and 53 were reported to have vomited. Variables used in the PSM method, as well as household demographics and water, sanitation, and hygiene characteristics stratified by case *versus* control status are presented in Table S1.

At least one of the three enteric viruses measured was found on 21% of mother’s hands (47/222) and in 3% of stored water samples (7/216). Rotavirus was the most frequently detected virus both on hands (10%) and in stored water (2%). Enterovirus was detected in 8% of hand samples, but was never detected in household stored water; adenovirus was detected in 5% of hand rinse samples and in 1% of stored water samples. BacHum was found in 39% of hand rinse samples (86/222) and in 14% of stored water samples (30/216).

More than half (59%) of all households in the study had at least one *E. coli* virulence gene (ECVG) detected in their stored water, and 41% of respondents had ECVG detected on their hands. The presence of individual virulence genes among all water and hand rinse samples processed for the case-control analysis is reported in Table S2. Across case and control households, the mean *E. coli* and enterococci concentration in household stored water was 1.5 (SD 1.0) and 0.5 (SD 0.6) log CFU/100 ml (N = 219), respectively. The mean *E. coli* and enterococci concentration in hand rinse samples was 2.5 (SD 1.0) and 2.7 (SD 1.0) log CFU/2 hands (N = 223), respectively. The concentration of FIB on hands and in stored drinking water stratified by case status can be found in Table S2. There was no significant difference between case and control households in the concentration of *E. coli* or enterococci found on mother’s hands or in stored drinking.

Table 1 presents the prevalence of molecular markers of enteric viruses and ECVG in household stored water and hand rinse samples, stratified by case and control households, as well as the odds ratios from the matched case-control analysis. The presence of ECVG in a household’s stored water was associated with a 2-fold *decrease* in the odds of at least one child under five years of age in the household reporting symptoms of diarrhea (OR = 0.51 [95% CI 0.27–0.93]; *p = *0.03). Similarly, the presence of the *E. coli* virulence gene, Lt1, on the primary caregiver’s hands (suggesting the presence of the *E. coli* pathotype enterotoxigenic *E. coli* (ETEC) [55]) was also associated with a significant decrease in the odds of a child in the household having diarrhea (OR = 0.25 [95% CI 0.05, 0.93]; *p = *0.04).

**Table 1:** Prevalence of *E. coli* virulence genes (ECVG), enteric virus genes, human-specific *Bacteroidales* genes, and FIB detected in household stored drinking water and hand rinse samples of respondents with at least one child younger than five years old that were either sick with diarrhea (cases) versus matched healthy children under five years of age (controls).

	HANDS							STORED WATER					
	Case (%)	Control (%)	OR	95% CI^c^		P		Case (%)	Control (%)	OR	95% CI^c^		P
ECVG a	40.0	44.1	0.86	0.48	1.53	0.68		52.7	67.9	0.51	0.27	0.93	0.03†
*ipaH*	20.0	31.5	0.50	0.24	1.01	0.05		23.2	33.9	0.58	0.31	1.07	0.09
*aggR*	13.6	15.3	0.92	0.39	2.19	1.00		21.4	32.1	0.54	0.26	1.07	0.08
*Lt1*	4.5	13.5	0.25	0.05	0.93	0.04†		11.6	17.0	0.71	0.31	1.57	0.46
*STIb*	0.0	0.9	1.00*	0.00	19.00	1.00		1.8	1.8	1.00	0.07	13.80	1.00
*eaeA*	4.5	5.4	0.83	0.20	3.28	1.00		17.0	15.2	1.15	0.51	2.64	0.85
*stx1*	9.1	17.1	0.53	0.22	1.19	0.14		20.5	30.4	0.54	0.26	1.07	0.08
*stx2*	0.0	0.9	1.00*	0.00	19.00	1.00		0.0	2.7	0.26*	0.00	1.71	0.25
Enteric Virus b	24.8	17.0	1.69	0.82	3.66	0.18		4.6	1.8	2.50	0.41	26.25	0.45
Rotavirus	12.4	8.0	1.56	0.63	4.07	0.40		3.7	0.0	5.29*	0.90	∞	0.13
Adenovirus	6.2	4.5	1.50	0.36	7.23	0.75		0.9	1.8	0.50	0.01	9.61	1.00
Enterovirus	8.8	6.3	1.60	0.46	6.22	0.58		0.0	0.0				
At least 1 enteric virus or ECVG	55.8	54.0	1.12	0.63	1.97	0.79		54.0	66.4	0.59	0.32	1.08	0.09
Human *Bacteroidales*	33.6	43.8	0.64	0.35	1.13	0.13		11.1	16.2	0.67	0.27	1.59	0.42
*Escherichia coli* ¥	81.4	74.3	1.44	0.76	2.80	0.29		81.4	87.6	0.63	0.28	1.37	0.28
1 to <11 CFU/100 mL								13.3	20.4	0.48	0.16	1.39	0.21
11 to 100 CFU/100 mL								31.9	33.6	0.70	0.25	1.85	0.57
>100 CFU/100 mL								36.3	33.6	0.79	0.29	2.04	0.75
*Enterococcus* ¥	84.1	87.6	0.71	0.28	1.73	0.54		79.6	89.4	0.48	0.20	1.06	0.07
1 to <11 CFU/100 mL								18.6	15.9	0.60	0.20	1.75	0.43
11 to 100 CFU/100 mL								31.0	38.1	0.47	0.19	1.10	0.09
>100 CFU/100 mL								30.1	35.4	0.46	0.17	1.16	0.11

The study consisted of 112 unique case households (containing 113 case children) and 111 unique households with only healthy children (containing 113 matched, control children).

^a^ At least one of the seven pathogenic *E. coli* virulence genes (ECVG) measured present.

^b^ At least one of the three enteric viruses measured (rotavirus, adenovirus, enterovirus) present.

^c^ CI, confidence interval.

Presence/Absence of CFU per 2 hands; Presence/Absence or within specified range of CFU/100 mL stored drinking water with 0 CFU/100 mL as the reference group.

Indicates a median unbiased estimate.

Statistically significant (p≤0.05).

There were no significant associations between virus detection in water or hand rinses and diarrhea case status in children. The presence of each enteric virus on hands and in stored water was consistently associated with increased odds of a child in the household having diarrhea (Table 1), although none achieved statistical significance. The presence of at least one enteropathogen molecular marker (enteric virus or ECVG) on hands or in water was also not significantly associated with diarrhea.

The presence of BacHum in either stored water or hand rinse samples was not associated with the odds of a child having diarrhea at the time of visit. Similarly, the presence of FIB (*E. coli* or enterococci) on hands or in stored water was not associated with cases of diarrhea. Higher concentrations of FIB in stored water (categorical variable: 1 to <11, 11 to 100, or >100 CFU/100 mL, Table 1) were also not associated with the odds of a child having diarrhea.

Case and control households were found to be similar with respect to the variables used in the PSM (Table S1, all *p*>0.05). Cases and controls had no statistically different characteristics (all *p*>0.05), with one exception: a larger percentage of control households had their stored drinking water covered (98% *versus* 89%, *p*<0.01). The results of the bivariate analyses performed as a robustness check and to control for other potential confounders in the case-control analysis can be found in the SI (Results S1 and Table S1). The revised odds ratios after adjusting for the variables significantly associated with case status are presented in Table S3. Since there were no substantive differences between the adjusted case-control analyses and the unadjusted analyses, the case-control results were considered robust. The incorporation of numerous matching variables in the PSM model may have overmatched households, causing the case-control effect estimates to be biased downward [61], [64], [65]. Therefore, the potential effect of overmatching on our results was assessed. The results of the overmatching analysis (see SI and Tables S4, S5, S6, and S7) show that matching case and control children on several control variables did not affect the results.

### Behavioral Determinants of Enteric Pathogen and Fecal Markers

We modeled ECVG, enteric virus, and BacHum presence in hand rinse samples and household stored water as a function of hygiene and water management behaviors. Household socioeconomic and demographic characteristics, as well as water supply, sanitation, and hygiene practices of all 306 households used in the models can be found in Table S8. Among the households modeled, twenty-five percent of households included an infant (<1 yr) present at the time of visit, with an average of 1.3 (SD 0.5) children under the age of five. The age of the primary caregivers in our study ranged from 16 to 68 years (median 25 yr). Households reported spending an average of $17 US (SD $9) per month per person. Only 15% of households had an on-plot water source, but 85% reported using an improved source as their main source of drinking water according to the WHO/UNICEF Joint Monitoring program definition [63] (*e.g.,* tap, borewell, or rainwater). A minority of households (16%) reported to have treated their stored water. At the time of sampling, mean storage time was 33 hours (SD 29), and 94% of stored water containers were observed to be covered. Thirty-eight percent of households had access to a sanitation facility with improved sanitation infrastructure, and 51% percent of households reported having a private sanitation facility.

The models of ECVG, enteric virus, and BacHum presence in hand rinse samples and household stored water are presented in Tables 2 and 3, respectively. We found a limited number of significant determinants of hand contamination. The liters *per capita* used per day for hand washing (natural-log transformed) was the only statistically significant explanatory hygiene behavior in the model of enteric virus presence on hands (*p*<0.01). Results indicate that doubling the quantity of water used *per capita* per day for hand washing was associated with a 2-fold decrease in the odds of a respondent having an enteric virus on her hands. In the model of ECVG presence on hands, the odds of detecting ECVG was approximately three times greater for those respondents who reported handling food or washing (both *p*<0.01) prior to sampling versus those reporting sitting. No hand hygiene behaviors were significant in the model of BacHum. It is important to note that our model explains only a small portion of the variation in microbial prevalence on hands, highlighting the unexplained variability in hand contamination within these communities.

**Table 2:** Binary logistic regression model of *E. coli* virulence genes (ECVG), enteric virus genes, and human-specific *Bacteroidales* genes presence in hand rinse samples as a function of hygiene behaviors.

	HANDS																	
	ECVG, N = 256*						Enteric Virus, N = 258*						Human-Specific *Bacteroidales,* N = 258*					
	pseudo R^2^ = 0.09, Max-rescaled R^2^ = 0.12						pseudo R^2^ = 0.06, Max-Rescaled R^2^ = 0.10						pseudo R^2^ = 0.03, Max-Rescaled R^2^ = 0.04					
	Likelihood Ratio: χ2 = 24.31, p<0.01						Likelihood Ratio: χ^2^ = 16.60, *p = *0.06						Likelihood Ratio: χ^2^ = 6.93, *p = *0.64					
**Variable**	**ß**	**SE**	**P**	**OR**	**95% CI**		**ß**	**SE**	**P**	**OR**	**95% CI**		**ß**	**SE**	**P**	**OR**	**95% CI**	
Intercept	−0.87	0.33	0.01				−1.43	0.37	0.00				−0.57	0.32	0.07			
Liters per capita per day used for hand washing^a^	−0.01	0.27	0.98	0.99	0.59	1.68	−1.00	0.34	0.00†	0.37	0.19	0.72	−0.15	0.26	0.56	0.86	0.51	1.43
Respondent washed hands within 1 h prior to sampling^b^	−0.08	0.30	0.77	0.92	0.51	1.64	−0.67	0.38	0.08	0.51	0.24	1.09	−0.13	0.29	0.66	0.88	0.50	1.56
Washing (clothes, dishes, hands, child) vs. Sittingb, c	1.04	0.41	0.01†	2.84	1.28	6.32	−0.22	0.56	0.69	0.80	0.27	2.39	0.05	0.41	0.91	1.05	0.47	2.34
Hand Washing vs. Sittingb, c	0.90	1.43	0.53	2.46	0.15	40.77	1.41	1.44	0.32	4.12	0.25	68.73	−13.76	894.20	0.99	<0.01	<0.01	>999.9
Food Preparation vs. Sittingb, c	1.23	0.34	0.00†	3.41	1.74	6.69	0.10	0.41	0.81	1.11	0.49	2.48	0.55	0.33	0.10	1.73	0.90	3.34
Other vs. Sittingb, c	−0.25	0.57	0.66	0.78	0.25	2.39	0.31	0.58	0.59	1.37	0.44	4.24	−0.38	0.56	0.49	0.68	0.23	2.04
Use of facility with improved sanitation infrastructure^b^	−0.30	0.29	0.30	0.74	0.43	1.30	0.16	0.34	0.64	1.17	0.60	2.26	0.29	0.28	0.30	1.34	0.78	2.30
Infant (<1 yr) present in household^b^	0.49	0.30	0.11	1.63	0.90	2.96	−0.27	0.37	0.47	0.77	0.37	1.58	−0.14	0.30	0.63	0.87	0.48	1.56
Regular monthly expenditures per person per 1000 TZS^d^	0.06	0.10	0.53	1.06	0.88	1.28	0.22	0.11	0.04	1.24	1.01	1.53	0.01	0.09	0.95	1.01	0.84	1.20

^a^ Ln-transformed.

^b^ Binary variables with values of 0 and 1.

^c^ Refers to the reported activity prior to the respondent having their hand rinse sample taken.

^d^ TZS Tanzanian Shillings.

N <306 because sample was lost or survey response not collected.

Statistically significant (p≤0.05).

**Table 3:** Binary logistic regression model of *E. coli* virulence genes (ECVG), enteric virus genes, and human-specific *Bacteroidales* gene presence in household stored water as a function of water management behaviors.

	STORED WATER																	
	ECVG, N = 276*						Enteric Virus, N = 267*						Human-Specific *Bacteroidales,* N = 267*					
	pseudo R^2^ = 0.06, Max-Rescaled R^2^ = 0.09						pseudo R^2^ = 0.11, Max-Rescaled R^2^ = 0.45						pseudo R^2^ = 0.13 Max-Rescaled R^2^ = 0.24					
	Likelihood Ratio: χ^2^ = 17.83, *p = *0.04						Likelihood Ratio: χ^2^ = 32.17, *p*<0.01						Likelihood Ratio: χ^2^ = 35.57 *p<*0.01					
**Variable**	**ß**	**SE**	**P**	**OR**	**95% CI**		**ß**	**SE**	**P**	**OR**	**95% CI**		**ß**	**SE**	**P**	**OR**	**95% CI**	
Intercept	1.34	0.89	0.13				−25.02	418.8	0.95				−3.89	1.38	0.00			
Stored water reportedly treatedb, c	−0.11	0.37	0.77	0.90	0.43	1.87	0.40	1.29	0.76	1.49	0.12	18.76	0.02	0.60	0.98	1.02	0.32	3.26
Time water stored in household (h)^a^	0.09	0.11	0.45	1.09	0.87	1.37	−0.41	0.36	0.25	0.66	0.33	1.34	0.47	0.25	0.06	1.60	0.99	2.60
Observed extraction method of stored water was riskyb, d	−0.04	0.38	0.92	0.96	0.46	2.03	12.16	285.8	0.97	1.90×10^6^	<0.01	>999.9	−0.17	0.56	0.77	0.85	0.28	2.56
Stored water reportedly collected from improved sourceb, e	−1.01	0.41	0.01†	0.36	0.16	0.82	11.01	306.1	0.97	6.02×10^4^	<0.01	>999.9	−1.69	0.45	0.00†	0.18	0.08	0.45
Water Fetching Time Per Day (min)^a^	0.03	0.15	0.87	1.03	0.77	1.37	−0.15	0.42	0.72	0.86	0.38	1.96	0.37	0.25	0.14	1.44	0.89	2.35
Modeled contamination present on hands of primary caregiver^b^	0.26	0.26	0.33	1.30	0.77	2.17	3.49	1.11	0.00†	32.7	3.68	290.42	1.28	0.43	0.00†	3.60	1.55	8.38
Use of facility with improved sanitation infrastructure^b^	−0.55	0.27	0.04†	0.58	0.34	0.98	−0.70	0.87	0.42	0.50	0.09	2.75	0.41	0.44	0.35	1.51	0.64	3.59
Infant (<1 yr) present in household^b^	−0.07	0.30	0.80	0.93	0.52	1.66	−11.96	243.5	0.96	0.00	<0.01	>999.9	−0.35	0.52	0.50	0.71	0.25	1.96
Regular monthly expenditures per person per 1000 TZS^f^	−0.08	0.09	0.37	0.92	0.77	1.10	−0.42	0.35	0.23	0.66	0.33	1.30	−0.05	0.14	0.75	0.95	0.72	1.26

^a^ Ln-transformed.

^b^ Binary variables with values of 0 and 1.

^c^ Boiling, chlorinating, filtering, or SODIS (versus no treatment including settling).

^d^ Cup, mug, or bowl (versus pouring, long handled dipper, or spigot).

^e^ Borewell, rainwater, or tap (versus shallow well, cart/tanker, surface water, or vendor).

^f^ TZS Tanzanian Shillings.

N <306 because sample was lost or survey response not collected.

Statistically significant (p≤0.05).

Contamination of stored water was associated with several water management variables. Stored drinking water collected from an improved source was associated with a 2.7-fold reduction in the odds of detecting ECVG contamination (*p* = 0.02) and a 5.6-fold decrease in the odds of detecting BacHum (*p*<0.01). Conversely, the odds of enteric virus and BacHum being present in a household’s stored water was 32.7 and 3.6 times greater for households where enteric viruses or BacHum were also present on the hands of the respondent (both *p*<0.01). Also, the use of a facility with improved sanitation infrastructure was associated with a 1.7-fold decrease in the odds of detecting ECVG in the household’s stored drinking water (*p* = 0.04).

## Discussion

The overall prevalence of enteropathogen genes and the human-specific *Bacteroidales* molecular marker in stored drinking water and hand rinse samples analyzed for this study (Table S2) was similar to that found in Bagamoyo, Tanzania by Mattioli *et al*. [33]. Other studies in Tanzania have also documented ECVG, enteric virus, and *Bacteroidales* molecular markers in soil, on household surfaces, on produce, and on hands in Tanzania [16], [47].

Unexpectedly, our study found that the presence of ECVG in stored water and on hands was associated with decreased odds of a child under the age of five having reported diarrhea–indicating a higher prevalence of ECVG among control households. One possible explanation for the observed negative association between ECVG presence and diarrhea is that an episode of child diarrhea in the household might have triggered efforts by the primary caregiver to improve stored drinking water quality, such as treatment or collection from an improved source. A greater percentage of respondents caring for children with diarrhea reported boiling their stored drinking water (16%) than those in control households (9%), although the difference did not reach statistical significance (p = 0.1). Notably, a prior study in Tanzania also found microbial water quality to be cleaner among households with child diarrhea [17].

Asymptomatic pathogen shedding among healthy control children could help explain the absence of association between the pathogens detected in the household environment and child diarrhea. A recent study to identify the etiology of child diarrhea in Tanzania found that 52% of healthy controls were infected with an enteric pathogen [53]. Asymptomatic shedding could be the result of subclinical infections, persistent shedding after illness symptoms have subsided, ingestion of pathogens below an infectious dose, or immunity developed by those children living in households with persistent pathogen contamination [66]–[69].

Several reported water management and hygiene behaviors were found to be significantly associated with molecular indicators of pathogen contamination in the household. For example, modeling the presence of enteric virus marker on hands suggests that the volume of water used for hand washing may be important in reducing enteric virus transmission. Currently, the World Health Organization does not recommend a minimum volume of water per person per day specifically for hand washing, with the assumption that this volume is dependent on level of service and water fetching distance [70]. Households in our study reported using an average of 1.8 L (SD = 1.3) per person per day for hand washing and had an average of 5.4 people (SD = 2.3) per household (Table S8). Therefore, for a household in our study to reduce their risk of viral hand contamination by 2-fold, each household would have to access an average of 19.3 extra L of water (3.6 L per person) per day for hand washing. This would be roughly equivalent to a household collecting one extra 20-L jerry can of water per day.

Respondents reporting activities involving washing soiled household items (such as clothes or dishes), and those who reported handling food immediately prior to sampling, were more likely to have ECVG detected on their hands. This may suggest, as proposed by others [71], that water used for washing might serve as a transport mechanism for bacterial pathogens within the home. Previous studies found Tanzanian fresh market produce to be contaminated with ECVG [47] and have detected increased levels of FIB on hands after handling produce or preparing food [16]. This may imply that bacterial pathogens could also be transferred from produce to hands and would explain the association between ECVG on hands and recent food handling observed in this study.

Stored drinking water collected from households using improved water sources was less likely to be contaminated with ECVG and BacHum. This result adds nuance to published evidence of significant post-supply FIB contamination of stored water, particularly stored water collected from improved sources [8], [14], [72], [73]. Together this suggests that while stored water may become re-contaminated with FIB after collection, improvements to water sources may still provide safer water at the point-of-use by preventing bacterial pathogens and human feces from entering drinking water prior to collection. Households with improved sanitation infrastructure also had reduced odds of ECVG detection in their stored water, suggesting that, like water sources, improvements to sanitation infrastructure (*e.g.,* the addition of a concrete slab, septic tank, or flush tank) may reduce the risk of pathogenic bacteria entering the household’s stored drinking water.

Stored water collected from the household of a respondent with BacHum detected on her hands was more likely to be contaminated with BacHum, implying that hands may be a source of human fecal contamination in stored drinking water. However, due to the cross-sectional study design, we cannot confirm the directionality of contamination. Interestingly, reported water treatment, storage time, use of a “risky” extraction method, and water fetching time were not found to be associated with markers of pathogen or human fecal presence in stored water. This result stands in contrast to other research which found higher FIB levels in drinking water of households that performed risky extraction methods [14] and lower FIB levels in households that actively treated stored water [15] or were served by on-site water sources [74]. However the results are consistent with previous research in Tanzania identifying fecal contamination on hands as the strongest predictor of fecal contamination levels in stored water [17]. Thus, the effects of safe water management behaviors may be offset or muted by contamination from hand contact.

Viral marker prevalence may have been too low in this study to detect significant associations with diarrhea or water management behaviors given our sample size. Also, the lower limit of detection of the viral assays ranged from 10^0^ to 10^2^ genomic units per 100 ml stored water or per two hands. Thus, health relevant concentrations below our detection limit could have been present but been undetected in our study [75]. Despite these limitations, viral prevalence trended toward an increase in the odds of a child having diarrhea in the household, and therefore should be prioritized for further research. Our findings suggest that research focused on child exposure to enteric pathogens in the household environment should include rotavirus, as it was the most prevalent virus found in both hand rinses and stored water of sample households. Rotavirus was also recently reported to be one of the most important etiological agents of childhood diarrhea in a multi-country, prospective case-control study [76].

The results presented herein should be interpreted in consideration of several study limitations. The cross-sectional design precludes determining the direction of effect between child diarrhea, behaviors, and exposure. Also, previous research has shown that microbial contamination of hands and drinking water can vary significantly over time [14], [16], and our cross sectional study design does not allow us to consider this variability. In addition, only nucleic acids of enteric pathogens were detected in this study; our methods do not characterize the infectivity or viability of the pathogens targeted. Diarrhea and many of the water management and hygiene behaviors used in our models were self-reported by the respondent; self-reported data has been found to introduce inaccuracy and bias into estimates of behavior [77]. Finally, we were unable to measure all potential enteric pathogens (*e.g.,* protozoa) [76] in this study, and it is possible that these unmeasured pathogens may have had a positive association with diarrhea.

Few studies have looked at enteric pathogen and human fecal molecular markers in household environments of low-income communities [16], [33], [47]; this study contributes new knowledge by examining the association between hygiene and water management behaviors and the presence of these markers. The identification of behaviors associated with molecular markers of enteric pathogens and human fecal contamination on hands and in water in Bagamoyo can be used to inform the development of more efficacious interventions aimed at reducing the burden of childhood diarrhea in other low-income communities. For example, our work suggests that increasing the quantity of water available for hand washing may reduce enteric virus transmission from hands. In addition, improvements in food hygiene practices and sanitation infrastructure may help alleviate pathogenic *E. coli* contamination within the home, while improved source water may prevent human fecal and pathogenic *E. coli* contamination of a household’s stored drinking water. To our knowledge, this is the first study to examine the association between child diarrhea and molecular markers of enteric pathogens in household stored water and hand rinses. Our results warrant further investigation into why stored drinking water was less frequently contaminated with bacterial pathogens in households with sick children. In combination, our analyses highlight the need to better understand the relative contribution and interdependence of household exposure routes to the burden of child diarrhea.

## Supporting Information

Table S1 **Child-level descriptive statistics from the case-control analysis.** The table includes variables used in the PSM, as well as household (HH) demographics, and water, sanitation, and hygiene characteristics by case and control status.
(DOCX)

Table S2 **ECVG, enteric virus gene, Human** ***Bacteroidales*** **gene, and FIB (** ***Escherichia coli*** **and** ***Enterococcus*** **) prevalence for households in the case-control study.**
(DOCX)

Table S3 **Adjusted, matched case-control analysis results.**
(DOCX)

Table S4 **Unmatched household case-control analysis results using original controls.**
(DOCX)

Table S5 **Unmatched household case-control analysis results using additional controls.**
(DOCX)

Table S6 **Prevalence (%) of ECVG, enteric virus genes, and Human** ***Bacteroidales*** **genes in additional control households.** The additional households, along with the original (case-control analysis) control households, were used in the robustness checks.
(DOCX)

Table S7 **Robustness checks logistic regression results.** These results were used to evaluate whether the propensity score is significantly associated with pathogen presence in the original (case-control analysis) control households.
(DOCX)

Table S8 **Household-level descriptive statistics from the water management and hygiene behavior models.** Household-level demographics, and water, sanitation, and hygiene characteristics of the households used in the logistic regression model of contamination as a function of water management and hygiene behaviors.
(DOCX)

Methods S1 **Detailed description of methods used for the overmatching analysis.**
(DOCX)

Results S1 **Results of robustness checks and overmatching analysis.**
(DOCX)
