# Supplementary material for: Enteric Pathogens in Stored Drinking Water and on Caregiver’s Hands in Tanzanian Households with and without Reported Cases of Child Diarrhea
Source: PLoS One. 2014 Jan 2;9(1):e84939. doi: 10.1371/journal.pone.0084939 (PMC3879350; doi:10.1371/journal.pone.0084939)
Supplement: Table S1 — Child-level descriptive statistics from the case-control analysis. The table includes variables used in the PSM, as well as household (HH) demographics, and water, sanitation, and hygiene characteristics by case and control status. (DOCX) [file pone.0084939.s001.docx]

Table S1. Child level descriptive statistics from the case-control analysis. The table includes variables used in the PSM, as well as household (HH) demographics, and water, sanitation, and hygiene characteristics by case and control status. N = 113 case children and 113 matched, control children. P-values are the results of the bivariate analyses performed as a robustness check and to determine variables that may be potential confounders in the case-control analysis.

|  | | **Study Group** | |  |
| --- | --- | --- | --- | --- |
| **Variable** | | **Case^*^** | **Control^*^** | ***P*** |
| *PSM Variables* | Mean (SD) child age (yr) | 2.0 (1.24) | 1.8 (1.2) | 0.22 |
|  | Mean No. (SD) of families in the housing unit ^¥, µ^ | 2.2 (1.9) | 2.3 (1.9) | 0.80 |
|  | No. (%) of HH located within urban area | 86 (76.1) | 90 (79.7) | 0.50 |
|  | No. (%) of HH where the mother/primary caregiver works outside the home | 31 (27.4) | 34 (30.1) | 0.66 |
|  | Median No. (IQR) of times mother reported hand washing with soap the previous day^¥^ | 2.0 (2.0) | 2.0 (3.0) | 0.37 |
|  | No. (%) of children with dirt observed on their palms | 46 (40.7) | 42 (37.2) | 0.59 |
|  | No. (%) of HH reporting the youngest child kid uses latrine regularly | 20 (17.7) | 18 (15.9) | 0.72 |
|  | No. (%) of HH that own their on plot water source (not including rain water) | 10 (8.9) | 7 (6.2) | 0.45 |
|  | Mean (SD) liters of water collected per capita per day | 37.6 (16.2) | 36.2 (15.0) | 0.50 |
|  | No. (%) of household’s reported main drinking water source type is a borewell | 30 (26.6) | 23 (20.4) | 0.27 |
| *Demographics* | Mean no. (SD) of children under 5 within the HH | 1.2 (0.5) | 1.3 (0.5) | 0.18 |
| *&* | No. (%) of HH with at least one infant (<1 yr) | 22 (19.5) | 33 (29.2) | 0.19 |
| *HH* | No. (%) of female children | 47 (41.6) | 59 (52.2) | 0.11 |
| *Characteristics* | No (%) of HH with electricity | 25 (22.3) | 25 (22.1) | 1.00 |
|  | No. (%) of HH with mobile phone service | 72 (64.3) | 73 (64.6) | 0.96 |
|  | No. (%) of literate mothers/primary caregivers | 88 (78.57) | 79 (69.9) | 0.14 |
|  | No. (%) of HH that own the home they live in (verses renting for cash, service, or employment) | 75 (66.4) | 68 (60.2) | 0.34 |
|  | Mean (SD) regular monthly expenditures per person (TZS) [US $ (SD), 2013] | 28618.8 (15020) [$18.01 ($9.45)] | 26710.71 (13449) [$16.81 ($8.46)] | 0.32 |
| *Hygiene* | Mean time (h) (SD) since respondent last wash their hands with soap | 3.1 (2.4) | 3.4 (3.5) | 0.50 |
|  | No. (%) of respondents who had washed their hands within 1 hour of hand rinse sample being taken | 30 (29.1) | 32 (32.0) | 0.66 |
|  | No. (%) of respondents whose activity prior to hand rinse sample was: |  |  | 0.27 |
|  | Sitting | 58 | 67 |  |
|  | Washing (clothes, dishes, hands, or child) | 20 | 11 |  |
|  | Food preparation | 24 | 27 |  |
|  | Other activity (gardening/farming or sweeping) | 10 | 7 |  |
|  | Mean log concentration (CFU/2 hands) (SD) of FIB in respondent's/primary caregiver's hand rinse |  |  |  |
|  | *Escherichia coli* | 2.6 (1.1) | 2.5 (1.0) | 0.29 |
|  | Enterococcus | 2.7 (1.0) | 2.8 (0.9) | 0.85 |
|  | Mean log turbidity (NTU) (SD) of mother's hand rinse | 1.3 (0.4) | 1.4 (0.4) | 0.37 |
|  | No. (%) of respondents/primary care givers with dirt observed on their palms | 19 (17.0) | 12 (10.7) | 0.18 |
|  | No. (%) of respondents/primary caregivers with dirt observed under their finger nails | 59 (52.7) | 53 (47.3) | 0.42 |
|  | No. (%) of children with dirt observed under their finger nails | 72 (63.7) | 74 (66.1) | 0.71 |
| *Sanitation* | No. (%) of HH with private sanitation | 52 (46.9) | 48 (44.0) | 0.68 |
|  | Median no. (IQR) of HH that share a sanitation facility | 1.0 (0-2) | 1.0 (0-3) | 0.49 |
|  | No. (%) of HH with roof on latrine that prevents rain | 24 (24.6) | 26 (25.4) | 0.83 |
|  | No. (%) of HH with latrine covered | 16 (15.4) | 15 (14.0) | 0.78 |
|  | No. (%) of HH whose sanitation facility has cement slab, septic tank, or flush tank | 39 (37.9) | 42 (39.3) | 0.84 |
| *Water* | No. (%) of HH reporting they treated the water currently stored in their home by:^∂^ | 21 (19.1) | 15 (13.4) | 0.25 |
|  | Boiling | 18 (15.9) | 10 (8.8) | 0.10 |
|  | Chlorinating | 0 (0.0) | 3 (2.7) | 0.25 |
|  | Cloth filtering | 6 (5.3) | 1 (0.9) | 0.06 |
|  | Settling | 4 (3.5) | 4 (3.5) | 1.00 |
|  | Other | 1 (0.9) | 1 (0.9) | 1.00 |
|  | Mean (SD) liters per capita per day (LPCD) of water used for:^µ^ |  |  |  |
|  | Drinking | 5.4 (2.4) | 5.3 (2.8) | 0.77 |
|  | Hand Washing | 1.8 (1.1) | 1.9 (1.5) | 0.93 |
|  | Bathing | 11.3 (5.5) | 10.2 (4.5) | 0.10 |
|  | Sanitation | 3.3 (2.4) | 2.9 (2.0) | 0.19 |
|  | Dishes | 3.2 (1.7) | 3.0 (1.7) | 0.34 |
|  | Mopping | 1.4 (1.9) | 1.3 (2.9) | 0.81 |
|  | Laundry | 11.8 (6.6) | 10.3 (5.6) | 0.05 |
|  | No. (%) of HH with stored water container covered (verses partially or not covered) at time of visit | 98 (89.1) | 111 (98.2) | 0.00 |
|  | No. (%) of HH whether the observed stored water extraction method by the respondent was “risky” (dipping a short-handled cup, mug, or bowl versus pouring, long handled dipper, or spigot) | 94 (85.5) | 96 (85.7) | 0.96 |
|  | Mean log concentration (CFU/100 ml) (SD) of FIB in HH stored water |  |  |  |
|  | *Escherichia coli* | 1.5 (1.1) | 1.5 (1.0) | 0.85 |
|  | Enterococcus | 1.4 (1.0) | 1.6 (0.9) | 0.09 |
|  | Mean log turbidity (NTU) (SD) of HH stored water | 0.5 (0.6) | 0.5 (0.7) | 0.51 |
|  | Mean time (min) (SD) spent collecting water per day^µ^ | 29.6 (34.0) | 30.1 (28.6) | 0.37 |
|  | Mean time (h) (SD) the water currently in the house has been stored^µ^ | 32.0 (25.8) | 34.2 (32.5) | 0.54 |
|  | No. (%) of HH reported main drinking water source type |  |  |  |
|  | Tap | 64 (56.6) | 65 (58.0) | 0.89 |
|  | Borewell | 30 (26.6) | 23 (20.5) | 0.27 |
|  | Shallow Well | 4 (3.5) | 8 (7.1) | 0.24 |
|  | Rainwater | 9 (8.0) | 2 (1.8) | 0.03 |
|  | Vendor | 5 (4.4) | 13 (11.6) | 0.08 |
|  | Surface Water | 1 (0.9) | 1 (0.9) | 1.00 |

SD, Standard Deviation

IQR, Interquartile Range

¥ Ln-transformed value used in the PSM

µ Values Ln-transformed to perform T-Test

∂ Some HH reported using more than one water treatment method

* N<113 because survey response not given. Percentages and statistics reflect sample size of non-missing values.
