# Supplementary material for: Enteric Pathogens in Stored Drinking Water and on Caregiver’s Hands in Tanzanian Households with and without Reported Cases of Child Diarrhea
Source: PLoS One. 2014 Jan 2;9(1):e84939. doi: 10.1371/journal.pone.0084939 (PMC3879350; doi:10.1371/journal.pone.0084939)
Supplement: Table S2 — ECVG, enteric virus gene, Human Bacteroidales gene, and FIB ( Escherichia coli and Enterococcus ) prevalence for households in the case-control study. (DOCX) [file pone.0084939.s002.docx]

Table S2. ECVG, enteric virus gene, Human *Bacteroidales* gene, and FIB (*Escherichia coli* and *Enterococcus*) prevalence for households in the case-control study.

|  | **Percentage of Samples Positive (%)** | |
| --- | --- | --- |
|  | **Stored Water** | **Hands** |
|  | **N = 221 / N = 216 / N = 223*^c^*** | **N = 218 / N = 222 / N = 223*^c^*** |
| ECVG*^a^* | 59.3 | 41.3 |
| *ipaH* | 28.5 | 25.2 |
| *aggR* | 26.7 | 14.2 |
| *Lt1* | 14.0 | 8.7 |
| *STIb* | 1.8 | 0.5 |
| *eaeA* | 16.3 | 4.6 |
| *stx1* | 25.3 | 13.3 |
| *stx2* | 1.4 | 0.5 |
| Enteric Virus*^b^* | 3.2 | 21.2 |
| Rotavirus | 1.9 | 10.4 |
| Adenovirus | 1.4 | 5.4 |
| Enterovirus | 0.0 | 7.7 |
| At least 1 enteric virus or ECVG | 62.0 | 56.2 |
| *Human Bacteroidales* | 13.9 | 38.7 |
| *Escherichia coli^¥^* | 84.3 | 77.6 |
| 1 to <11 CFU/100mL | 17.0 |  |
| 11 to 100 CFU/100mL | 32.3 |  |
| >100 CFU/100mL | 35.0 |  |
| *Enterococcus^¥^* | 84.3 | 85.7 |
| 1 to <10 CFU/100mL | 17.5 |  |
| 11 to <100 CFU/100mL | 34.5 |  |
| >100 CFU/100mL | 32.3 |  |

*a* At least one of the seven pathogenic *E. coli* virulence genes (ECVG) measured present

*b* At least one of the three enteric viruses measured (rotavirus, adenovirus, enterovirus) present

*c* N = Number of *E. coli* virulence gene (ECVG) samples / N = Number of viral and BacHum samples / N = Number of FIB samples. N < 223 in some cases because samples were lost.

¥ Presence/Absence of CFU per 2 hands; Presence/Absence or within specified range of CFU/100 ml stored drinking water with 0 CFU/100ml as the reference group
