# Supplementary material for: Enteric Pathogens in Stored Drinking Water and on Caregiver’s Hands in Tanzanian Households with and without Reported Cases of Child Diarrhea
Source: PLoS One. 2014 Jan 2;9(1):e84939. doi: 10.1371/journal.pone.0084939 (PMC3879350; doi:10.1371/journal.pone.0084939)
Supplement: Table S3 — Adjusted, matched case-control analysis results. (DOCX) [file pone.0084939.s003.docx]

Table S3. Adjusted, matched case-control analysis results. N = 113 case children and N = 113 control children.

|  | **HANDS** | | | |  | **STORED WATER** | | | |
| --- | --- | --- | --- | --- | --- | --- | --- | --- | --- |
|  | **AOR** | **95% CI^c^** | | **P** |  | **AOR** | **95% CI^c^** | | **P** |
| ECVG*^a^* | 0.88 | 0.47 | 1.66 | 0.79 |  | 0.52 | 0.27 | 0.98 | 0.04^†^ |
| *ipaH* | 0.53 | 0.23 | 1.14 | 0.11 |  | 0.60 | 0.30 | 1.14 | 0.13 |
| *aggR* | 0.56 | 0.20 | 1.49 | 0.29 |  | 0.49 | 0.22 | 1.01 | 0.05 |
| *Lt1* | 0.26 | 0.05 | 0.98 | 0.05^†^ |  | 0.76 | 0.32 | 1.74 | 0.60 |
| *STIb* | 1.00^*^ | 0.00 | 19.00 | 1.00 |  | 1.00 | 0.07 | 13.80 | 1.00 |
| *eaeA* | 0.98 | 0.23 | 4.00 | 1.00 |  | 1.13 | 0.49 | 2.67 | 0.91 |
| *stx1* | 0.62 | 0.24 | 1.49 | 0.33 |  | 0.67 | 0.31 | 1.38 | 0.31 |
| *stx2* | 1.00^*^ | 0.00 | 19.00 | 1.00 |  | 0.08^*^ | 0.00 | 0.96 | 0.09 |
| Enteric Virus^b^ | 1.32 | 0.58 | 3.10 | 0.59 |  | 3.56 | 0.48 | 48.63 | 0.30 |
| Rotavirus | 1.39 | 0.51 | 4.05 | 0.64 |  | 4.67^*^ | 0.78 | ∞ | 0.16 |
| Adenovirus | 1.73 | 0.35 | 9.77 | 0.66 |  | 0.88 | 0.01 | 25.48 | 1.00 |
| Enterovirus | 1.27 | 0.31 | 5.76 | 0.95 |  |  |  |  |  |
| At least 1 enteric virus or ECVG | 0.997 | 0.54 | 1.833 | 1.00 |  | 0.60 | 0.31 | 1.13 | 0.12 |
| *Human Bacteroidales* | 0.71 | 0.37 | 1.32 | 0.31 |  | 0.60 | 0.23 | 1.48 | 0.32 |
| *Escherichia coli^¥^* | 1.60 | 0.79 | 3.38 | 0.22 |  | 0.60 | 0.24 | 1.44 | 0.29 |
| 1 to <11 CFU/100mL |  |  |  |  |  | 0.42 | 0.13 | 1.31 | 0.15 |
| 11 to 100 CFU/100mL |  |  |  |  |  | 0.68 | 0.25 | 1.66 | 0.42 |
| >100 CFU/100mL |  |  |  |  |  | 0.63 | 0.23 | 1.66 | 0.42 |
| *Enterococcus^¥^* | 0.58 | 0.21 | 1.52 | 0.32 |  | 0.51 | 0.20 | 1.20 | 0.14 |
| 1 to <10 CFU/100mL |  |  |  |  |  | 0.67 | 0.21 | 2.09 | 0.61 |
| 11 to <100 CFU/100mL |  |  |  |  |  | 0.54 | 0.20 | 1.34 | 0.21 |
| >100 CFU/100mL |  |  |  |  |  | 0.44 | 0.15 | 1.17 | 0.11 |

a At least one of the seven pathogenic *E. coli* virulence genes (ECVG) measured present

b At least one of the three enteric virus genes measured (rotavirus, adenovirus, enterovirus) present

c CI, confidence interval

¥ Presence/Absence of CFU per 2 hands; Presence/Absence or within specified range of CFU/100 ml stored drinking water with 0 CFU/100ml as the reference group

* Indicates a median unbiased estimate

^†^ Statistically significant (p ≤ 0.05)
