# Supplementary material for: Enteric Pathogens in Stored Drinking Water and on Caregiver’s Hands in Tanzanian Households with and without Reported Cases of Child Diarrhea
Source: PLoS One. 2014 Jan 2;9(1):e84939. doi: 10.1371/journal.pone.0084939 (PMC3879350; doi:10.1371/journal.pone.0084939)
Supplement: Table S4 — Unmatched household case-control analysis results using original controls. (DOCX) [file pone.0084939.s004.docx]

Table S4. Unmatched HH case-control analysis results using original controls. N = 112 case households and N = 111 control households.

|  | **HANDS** | | | | **STORED WATER** | | | |
| --- | --- | --- | --- | --- | --- | --- | --- | --- |
|  | **OR** | **95% CI^c^** | | **P** | **OR** | **95% CI^c^** | | **P** |
| ECVG*^a^* | 0.83 | 0.47 | 1.47 | 0.58 | 0.53 | 0.30 | 0.95 | 0.03^†^ |
| *ipaH* | 0.53 | 0.27 | 1.03 | 0.06 | 0.61 | 0.32 | 1.14 | 0.13 |
| *aggR* | 0.93 | 0.40 | 2.13 | 1.00 | 0.59 | 0.31 | 1.13 | 0.12 |
| *Lt1* | 0.33 | 0.09 | 1.01 | 0.05^†^ | 0.64 | 0.27 | 1.45 | 0.33 |
| *STIb* | 1.00^*^ | 0.00 | 19.00 | 1.00 | 0.99 | 0.07 | 13.90 | 1.00 |
| *eaeA* | 0.83 | 0.19 | 3.33 | 1.00 | 1.13 | 0.52 | 2.47 | 0.88 |
| *stx1* | 0.48 | 0.19 | 1.15 | 0.11 | 0.55 | 0.28 | 1.07 | 0.08 |
| *stx2* | 1.00^*^ | 0.00 | 19.00 | 1.00 | 0.25^*^ | 0.00 | 1.69 | 0.24 |
| Enteric Virus^b^ | 1.59 | 0.79 | 3.26 | 0.21 | 2.61 | 0.42 | 28.01 | 0.43 |
| Rotavirus | 1.60 | 0.61 | 4.40 | 0.40 | 5.52^*^ | 0.93 | ∞ | 0.12 |
| Adenovirus | 1.40 | 0.37 | 5.77 | 0.79 | 0.51 | 0.01 | 9.86 | 1.00 |
| Enterovirus | 1.44 | 0.47 | 4.64 | 0.64 |  |  |  |  |
| At least 1 enteric virus or ECVG | 1.06 | 0.60 | 1.87 | 0.95 | 0.61 | 0.33 | 1.09 | 0.10 |
| Human *Bacteroidales* | 0.67 | 0.37 | 1.18 | 0.18 | 0.64 | 0.27 | 1.50 | 0.35 |

a At least one of the seven pathogenic *E. coli* virulence genes (ECVG) measured present

b At least one of the three enteric virus genes measured (rotavirus, adenovirus, enterovirus) present

c CI, confidence interval

* Indicates a median unbiased estimate

^†^ Statistically significant (p ≤ 0.05)
