# Supplementary material for: Enteric Pathogens in Stored Drinking Water and on Caregiver’s Hands in Tanzanian Households with and without Reported Cases of Child Diarrhea
Source: PLoS One. 2014 Jan 2;9(1):e84939. doi: 10.1371/journal.pone.0084939 (PMC3879350; doi:10.1371/journal.pone.0084939)
Supplement: Table S5 — Unmatched household case-control analysis results using additional controls. (DOCX) [file pone.0084939.s005.docx]

Table S5. Unmatched HH case-control analysis results using additional controls. N = 112 case households and N = 158 control households.

|  | **HANDS** | | | | **STORED WATER** | | | |
| --- | --- | --- | --- | --- | --- | --- | --- | --- |
|  | **OR** | **95% CI^c^** | | **P** | **OR** | **95% CI^c^** | | **P** |
| ECVG *^a^* | 0.89 | 0.52 | 1.51 | 0.74 | 0.59 | 0.35 | 1.00 | 0.05^†^ |
| *ipaH* | 0.65 | 0.34 | 1.21 | 0.19 | 0.59 | 0.32 | 1.04 | 0.07 |
| *aggR* | 0.84 | 0.39 | 1.75 | 0.75 | 0.73 | 0.39 | 1.34 | 0.35 |
| *Lt1* | 0.42 | 0.12 | 1.26 | 0.14 | 0.70 | 0.31 | 1.51 | 0.43 |
| *STIb* | 1.43^*^ | 0.00 | 27.19 | 1.00 | 0.70 | 0.06 | 5.00 | 1.00 |
| *eaeA* | 0.79 | 0.20 | 2.70 | 0.90 | 1.04 | 0.51 | 2.09 | 1.00 |
| *stx1* | 0.53 | 0.22 | 1.21 | 0.15 | 0.56 | 0.30 | 1.03 | 0.07 |
| *stx2* | 0.59^*^ | 0.00 | 4.97 | 0.69 | 0.26^*^ | 0.00 | 1.57 | 0.23 |
| Enteric Virus ^b^ | 1.52 | 0.81 | 2.88 | 0.21 | 2.48 | 0.47 | 16.29 | 0.37 |
| Rotavirus | 1.71 | 0.70 | 4.24 | 0.27 | 5.94^*^ | 0.58 | 296.39 | 0.18 |
| Adenovirus | 1.23 | 0.37 | 4.02 | 0.89 | 0.72 | 0.01 | 14.04 | 1.00 |
| Enterovirus | 1.29 | 0.47 | 3.49 | 0.73 |  |  |  |  |
| At least 1 enteric virus or ECVG | 1.16 | 0.69 | 1.96 | 0.65 | 0.65 | 0.38 | 1.11 | 0.12 |
| Human *Bacteroidales* | 0.74 | 0.43 | 1.26 | 0.29 | 0.81 | 0.34 | 1.81 | 0.72 |

a At least one of the seven pathogenic *E. coli* virulence genes (ECVG) measured present

b At least one of the three enteric virus genes measured (rotavirus, adenovirus, enterovirus) present

c CI, confidence interval

* Indicates a median unbiased estimate

^†^ Statistically significant (p ≤ 0.05)
