# Supplementary material for: Enteric Pathogens in Stored Drinking Water and on Caregiver’s Hands in Tanzanian Households with and without Reported Cases of Child Diarrhea
Source: PLoS One. 2014 Jan 2;9(1):e84939. doi: 10.1371/journal.pone.0084939 (PMC3879350; doi:10.1371/journal.pone.0084939)
Supplement: Table S6 — Prevalence (%) of ECVG, enteric virus genes, and Human Bacteroidales genes in additional control households. The additional households, along with the original (case-control analysis) control households, were used in the robustness checks. (DOCX) [file pone.0084939.s006.docx]

Table S6. Prevalence (%) of ECVG, enteric virus genes, and Human *Bacteroidales* genes in additional control households. The additional households, along with the original case-control analysis, control households, were used in the robustness checks.

|  | **Additional Control households (N = 47)** | | | | **Original Control households (N = 111)** | |
| --- | --- | --- | --- | --- | --- | --- |
|  | **Stored Water** | **P^*^** | **Hands** | **P^*^** | **Stored Water** | **Hands** |
| ECVG | 58.3% | 0.33 | 39.6% | 0.68 | 66.4% | 43.1% |
| *ipaH* | 35.4% | 0.83 | 18.8% | 0.11 | 33.6% | 31.2% |
| *aggR* | 16.7% | 0.05^†^ | 18.8% | 0.53 | 31.8% | 14.7% |
| *Lt1* | 12.5% | 0.53 | 4.2% | 0.10 | 16.4% | 12.8% |
| *STIb* | 4.2% | 0.39 | 0.0% | 0.51 | 1.8% | 0.9% |
| *eaeA* | 18.8% | 0.61 | 6.3% | 0.66 | 15.5% | 4.6% |
| *stx1* | 29.2% | 0.83 | 12.5% | 0.44 | 30.9% | 17.4% |
| *stx2* | 2.1% | 0.81 | 2.1% | 0.55 | 2.7% | 0.9% |
| Enteric Virus | 2.1% | 0.90 | 19.1% | 0.78 | 1.8% | 17.3% |
| Rotavirus | 2.1% | 0.30 | 6.4% | 1.00 | 0.0% | 8.2% |
| Adenovirus | 0.0% | 1.00 | 6.4% | 0.70 | 1.8% | 4.5% |
| Enterovirus | 0.0% | - | 0.1% | 0.73 | 0.0% | 6.4% |
| *Human Bacteroidales* | 8.5% | 0.19 | 34.0% | 0.26 | 16.5% | 43.6% |

a At least one of the seven pathogenic *E. coli* virulence genes (ECVG) measured present

b At least one of the three enteric virus genes measured (rotavirus, adenovirus, enterovirus) present

* P-values represent statistical difference between prevalence in additional control households and prevalence in original case-control, control study (chi-square test).

^†^ Statistically significant (p ≤ 0.05)
