# Supplementary material for: Enteric Pathogens in Stored Drinking Water and on Caregiver’s Hands in Tanzanian Households with and without Reported Cases of Child Diarrhea
Source: PLoS One. 2014 Jan 2;9(1):e84939. doi: 10.1371/journal.pone.0084939 (PMC3879350; doi:10.1371/journal.pone.0084939)
Supplement: Table S7 — Robustness checks logistic regression results. These results were used to evaluate whether the propensity score is significantly associated with pathogen presence in the original (case-control analysis) control households. (DOCX) [file pone.0084939.s007.docx]

Table S7. Robustness checks logistic regression results. These results were used to evaluate whether the propensity score is significantly associated with pathogen presence in original case-control analysis, control households (N = 111 control households).

|  | **HANDS** | | | **STORED WATER** | | |
| --- | --- | --- | --- | --- | --- | --- |
|  | **ß^*^** | **OR** | **P** | **ß^*^** | **OR** | **P** |
| ECVG*^a^* | 2.79 | 16.32 | 0.04^†^ | -2.61 | 0.07 | 0.07 |
| *ipaH* | 2.03 | 7.60 | 0.15 | -1.50 | 0.22 | 0.30 |
| *aggR* | 2.48 | 11.90 | 0.15 | -1.45 | 0.23 | 0.32 |
| *Lt1* | -1.22 | 0.30 | 0.55 | -0.56 | 0.57 | 0.76 |
| *STIb* | 1.22 | 3.40 | 0.85 | -1.74 | 0.18 | 0.75 |
| *eaeA* | 7.19 | 1321.21 | 0.01^†^ | -1.53 | 0.22 | 0.44 |
| *stx1* | -1.07 | 0.34 | 0.56 | -1.99 | 0.14 | 0.19 |
| *stx2* | 3.88 | 48.44 | 0.50 | -13.56 | 0.00 | 0.15 |
| Enteric Virus^b^ | 0.47 | 1.59 | 0.79 | 3.20 | 24.62 | 0.45 |
| Rotavirus | 1.91 | 6.73 | 0.39 | - | - | - |
| Adenovirus | -0.59 | 0.56 | 0.86 | 3.20 | 24.62 | 0.45 |
| Enterovirus | 1.28 | 3.61 | 0.62 | - | - | - |
| Human *Bacteroidales* | -0.06 | 0.94 | 0.96 | -4.96 | 0.01 | 0.03 |

a At least one of the seven pathogenic *E. coli* virulence genes (ECVG) measured present

b At least one of the three enteric virus genes measured (rotavirus, adenovirus, enterovirus) present

(-): Analysis was not possible due to lack of viral gene presence in sample type

^†^ Statistically significant (p ≤ 0.05)

^*^ Non-standardized beta coefficient
