# Supplementary material for: Enteric Pathogens in Stored Drinking Water and on Caregiver’s Hands in Tanzanian Households with and without Reported Cases of Child Diarrhea
Source: PLoS One. 2014 Jan 2;9(1):e84939. doi: 10.1371/journal.pone.0084939 (PMC3879350; doi:10.1371/journal.pone.0084939)
Supplement: Table S8 — Household-level descriptive statistics from the water management and hygiene behavior models. Household-level demographics, and water, sanitation, and hygiene characteristics of the households used in the logistic regression model of contamination as a function of water management and hygiene behaviors. (DOCX) [file pone.0084939.s008.docx]

**Table S8.** **Household level descriptive statistics from the water management and hygiene behavior models.** Household (HH) demographics, and water, sanitation, and hygiene characteristics of the 306 HH used in logistic regression model of contamination as a function of water management and hygiene behaviors.

|  | **Variable** | **N = 306^*^ HH** |
| --- | --- | --- |
| *Demographics* | Mean no. (SD) of children under 5 within the HH | 1.26 (0.5) |
|  | No. (%) of HH with at least one infant (<1 yr) | 78 (25.5) |
|  | Mean No. (SD) of families in the housing unit | 2.1 (1.9) |
|  | No. (%) of HH located within urban (verses rural community) | 98 (32.1) |
|  | No (%) of HH with electricity | 69 (22.6) |
|  | No. (%) of HH with mobile phone service | 193 (63.3) |
|  | No. (%) of literate mothers/primary caregivers | 232 (76.1) |
|  | No. (%) of HH where the mother/primary caregiver works outside the home | 80 (26.2) |
|  | No. (%) of HH that own the home they live in (verses renting for cash, service, or employment) | 204 (66.9) |
|  | Mean (SD) regular monthly expenditures per person (TZS) [US $ (SD), 2013] | 28131.8 (15083) [17.4 (9.3)] |
| *Hygiene* | Mean No. (SD) of times mother reported hand washing with soap the previous day | 2.7 (2.2) |
|  | No. (%) of respondents reported hand washing within 1 hour prior to hand rinse sampling | 84 (30.4) |
|  | No. (%) of respondents reported activity prior to hand rinse sampling: |  |
|  | Sitting | 177 (58.2) |
|  | Washing (clothes, dishes, child) | 38 (12.5) |
|  | Hand washing | 3 (1.0) |
|  | Food preparation/eating/serving | 66 (21.7) |
|  | Other activity (gardening/farming or sweeping) | 20 (6.6) |
|  | Mean time (h) (SD) since respondent last wash their hands with soap | 3.2 (2.9) |
|  | Mean Log concentration (CFU/2 hands) (SD) of FIB on mother's hands: |  |
|  | *Escherichia coli* | 2.5 (1.0) |
|  | Enterococcus | 2.7 (1.0) |
|  | Mean log turbidity (NTU) (SD) of mother's hand rinse | 1.3 (0.4) |
|  | No. (%) of respondents/primary care givers with dirt observed on their palms | 263 (86.5) |
|  | No. (%) of respondents/primary care givers with dirt observed under their finger nails | 154 (50.7) |
| *Sanitation* | No. (%) of HH that have a private sanitation | 153 (51.0) |
|  | Mean no. (SD) of HH that share a sanitation facility | 1.3 (1.8) |
|  | No. (%) of HH with latrine covered | 240 (82.8) |
|  | No. (%) of HH whose sanitation facility has cement slab, septic tank, or flush tank | 110 (37.9) |
|  | No. (%) of HH reporting using diapers/cloth pieces in the home | 142 (46.6) |
|  | No. (%) of HH reporting small children using a potty chair regularly | 88 (28.9) |
|  | No. (%) of HH reporting young children defecating on the grounds | 177 (58.0) |
|  | No. (%) of HH reporting the youngest child kid uses latrine regularly | 55 (18.0) |
| *Water* | No. (%) of HH that own their on plot water source (not including rain water) | 47 (15.4) |
|  | No. (%) of HH reporting they treated the water currently stored in their home by:^∂^ | 48 (15.9) |
|  | Boiling | 37 |
|  | Chlorinating | 3 |
|  | Cloth filtering | 10 |
|  | Settling | 8 |
|  | Other | 2 |
|  | Mean (SD) liters per capita per day (LPCD) used for: | 35.8 (14.5) |
|  | Drinking | 5.2 (2.6) |
|  | Hand Washing | 1.8 (1.3) |
|  | Bathing | 10.8 (4.8) |
|  | Sanitation | 3.0 (2.3) |
|  | Dishes | 3.1 (1.7) |
|  | Mopping | 1.4 (2.3) |
|  | Laundry | 10.5 (6.0) |
|  | No. (%) of HH with stored water container covered (versus partially or not covered) at time of visit | 286 (94.4) |
|  | No. (%) of HH whether the observed stored water extraction method by the respondent was “risky” (dipping a short-handled cup, mug, or bowl versus pouring, long handled dipper, or spigot) | 258 (85.7) |
|  | Mean log concentration (CFU/100 mL) (SD) of FIB in HH stored water: |  |
|  | *Escherichia coli* | 1.5 (1.0) |
|  | Enterococcus | 1.5 (0.9) |
|  | Mean log turbidity (NTU) (SD) of HH stored water | 0.5 (0.7) |
|  | Mean time (min) (SD) spent collecting water per day | 30.0 (37.1) |
|  | Mean time (h) (SD) the water currently in the house has been stored | 32.6 (28.9) |
|  | No. (%) HH reported main drinking water source type: |  |
|  | Tap | 184 (60.5) |
|  | Borewell | 56 (18.4) |
|  | Shallow Well | 24 (7.9) |
|  | Rainwater | 17 (5.6) |
|  | Vendor | 19 (6.3) |
|  | Surface Water | 1 (0.3) |

SD, Standard Deviation

IQR, Interquartile Range

∂ Some HH reported using more than one water treatment method

* N<306 because survey response not given. Percentages and statistics reflect sample size of non-missing values.
