# Supplementary material for: Enteric Pathogens in Stored Drinking Water and on Caregiver’s Hands in Tanzanian Households with and without Reported Cases of Child Diarrhea
Source: PLoS One. 2014 Jan 2;9(1):e84939. doi: 10.1371/journal.pone.0084939 (PMC3879350; doi:10.1371/journal.pone.0084939)
Supplement: Methods S1 — Detailed description of methods used for the overmatching analysis. (DOCX) [file pone.0084939.s009.docx]

**METHODS**

*Overmatching analysis*

The incorporation of numerous matching variables in the Propensity Score Matching (PSM) model may have overmatched households, causing the case-control effect estimates to be biased downward [1-3]. Therefore, the potential effect of overmatching on our results was assessed. To examine the potential for overmatching in the case-control analysis, several robustness checks were performed. First, the data were re-analyzed unmatched using chi-squared tests. Next, the data were re-analyzed with an additional set of control households (N = 47) using unmatched chi-square tests [3, 4]. The additional controls were households from the same study population that fit the control criteria of the present study [5]. Finally, the control households matched to cases by PSM were compared to the unmatched additional control households to determine: 1) if the exposures of interest are statistically different in between the two control groups (indicating if the matched control group is representative of a random sample from the study population) using a chi-square test; and 2) if the propensity score is strongly associated with the presence of contamination among the control group using logistic regression.

**REFERENCES**

1. Day NE, Byar DP and Green SB (1980) Overadjustment in case-control studies. Am J Epidemiol 112: 696-706.
2. Wacholder S, Silverman DT, McLaughlin JK and Mandel JS (1992) Selection of controls in case-control studies. III. Design options. Am J Epidemiol 135: 1042-1050.
3. Breslow NE, Day NE and Cancer IAfRo (1980) Statistical methods in cancer research. vol. 1: The analysis of case-control studies.
4. Ibrahim MA and Spitzer WO (1979) The case control study: the problem and the prospect. Journal of chronic diseases 32: 139-144.
5. Mattioli MC, Pickering AJ, Gilsdorf RJ, Davis J and Boehm AB (2013) Hands and water as vectors of diarrheal pathogens in Bagamoyo, Tanzania. Environ Sci Technol 47: 355-363.
