# Supplementary material for: Enteric Pathogens in Stored Drinking Water and on Caregiver’s Hands in Tanzanian Households with and without Reported Cases of Child Diarrhea
Source: PLoS One. 2014 Jan 2;9(1):e84939. doi: 10.1371/journal.pone.0084939 (PMC3879350; doi:10.1371/journal.pone.0084939)
Supplement: Results S1 — Results of robustness checks and overmatching analysis. (DOCX) [file pone.0084939.s010.docx]

**RESULTS SI**

*Robustness analysis*

Below are the detailed results from the bivariate analyses of household demographics and water, sanitation, and hygiene behaviors/characteristics between case and control households. The bivariate analyses revealed that six household demographic/behavioral variables were different in households with diarrhea compared to households without diarrhea (*p* < 0.20): child gender, absence of an infant (<1 yr) in the household, the respondent being able to both read and write, presence of dirt on the respondent’s palms, the stored drinking water being partially covered or not covered at the time of visit, and fewer children in the household under the age of five (Table S1). In the reduced form of the multivariate logistic model of diarrhea, two variables remained statistically significantly associated with cases of diarrhea in our study households (*p* ≤ 0.05): whether the stored water fully was covered (adjusted OR (AOR) = 0.105 [95% CI, 0.021, 0.523]; *p* = 0.006) and the number of children under the age of five in the household (AOR = 0.537 [95% CI, 0.298, 0.970]; *p* = 0.039).

*Overmatching analysis*

The overmatching analysis results can be found in Tables S4-S7. No additional significant associations were found in the unmatched analysis with the original study control households or with the additional control households. The virulence gene, *Lt1*, on hands was not significantly associated with a child having diarrhea in either unmatched analyses. The virulence gene, *aggR*, was present in a significantly greater percentage (*p* = 0.049) of stored water samples from the original control households (31.8%) compared to the additional control households (16.7%). However, ECVG and the virulence gene, *eaeA*, on hands were significantly associated with the propensity score assigned to a household (ß = 2.79, *p* = 0.044; ß = 7.19, *p* = 0.014), and the presence of BacHum in a household stored water was also significantly associated with the propensity score (ß = -4.96, *p* = 0.033).
